# Determination of Acrylamide in Foods by Liquid Chromatography–Tandem Mass Spectrometry with a Porous Graphitic Carbon Column

**DOI:** 10.3390/molecules31213981

**Published:** 2026-11-04

**Authors:** Huijiao Chen, Lingang Chen, Weiping Xie

**Affiliations:** 1 Department of Physical and Chemical Laboratory, Quanzhou Center for Disease Control and Prevention (Quanzhou Health Supervision Institute), Quanzhou 362000, China; chenhj_10@163.com (H.C.); 15260792780@163.com (L.C.); 2 The Affiliated Quanzhou Center for Disease Control and Prevention of Fujian Medical University, Quanzhou 362000, China

**Keywords:** acrylamide, porous graphitic carbon column, liquid chromatography–tandem mass spectrometry, food safety, matrix effect

## Abstract

A high-performance liquid chromatography–tandem mass spectrometry (HPLC–MS/MS) method was established for the determination of acrylamide in foods using a porous graphitic carbon (PGC) column for separation. Samples were spiked with an isotopically labeled internal standard, extracted with acetonitrile–water, purified using a mixed-mode cation-exchange (MCX) solid-phase extraction cartridge, and then separated on a PGC column. By employing a single-step pass-through MCX clean-up, the inherent anti-interference capacity of the PGC column in complex matrices was evaluated. Methanol and 0.1% formic acid in water served as the mobile phase, and detection was performed in positive electrospray ionization mode with multiple reaction monitoring (MRM). Method validation was conducted using meat products and coffee samples, and the method was further applied to eight sample categories (*n* = 68), including potato chips, biscuits, instant coffee, and local specialty foods from southern Fujian. Acrylamide exhibited good linearity over the concentration range of 10–500 µg/L, with correlation coefficients of 0.9994–0.9998. The limit of detection (LOD) was 0.81–1.38 µg/kg, and the limit of quantification (LOQ) was 2.70–4.61 µg/kg. The mean spiked recovery was 94.2–103.4%, with a relative standard deviation (RSD) of 0.8–5.4%, and the matrix effect was 60.6–76.5% for the PGC column, compared with 46.8–51.3% for the C_18_ column. A paired *t*-test showed that the PGC column exhibited a significantly higher matrix effect (ME) value (i.e., less signal suppression) than the C_18_ column (*p* < 0.05). Owing to the strong retention characteristics of the PGC column for polar compounds, the method effectively improves chromatographic separation and reduces co-eluting interferences. It provides a practical approach for reducing matrix interference and improving sensitivity in matrices where interferences are well-controlled (e.g., meat products), while effectively overcoming the overestimation of acrylamide in highly complex matrices like coffee, with a residual matrix effect corrected by the isotope-labeled internal standard specifically within these validated matrices.

## 1. Introduction

Acrylamide (AA) is a small-molecule organic compound with the molecular formula C_3_H_5_NO, primarily used as a monomer in the synthesis of polyacrylamides [1]. In April 2002, the Swedish National Food Administration, in collaboration with Stockholm University, first reported the presence of relatively high levels of AA in heat-treated potato products and other baked foods [2]. This finding attracted widespread global attention. AA is generally considered to originate from the Maillard reaction between reducing sugars and free asparagine, which serves as the primary amino acid precursor [3,4]. Thermal processing is indispensable in modern food processing. During high-temperature treatments (typically above 120 °C) such as frying and baking, foods rich in reducing sugars and free asparagine develop characteristic color and flavor through the Maillard reaction [5]. As a by-product of this reaction, AA in food has attracted extensive attention because of its potential health risks [6]. AA exhibits neurotoxicity, immunotoxicity, carcinogenicity, and other harmful effects [7,8], and the International Agency for Research on Cancer (IARC) classifies AA as a Group 2A carcinogen [9]. Therefore, accurate analysis of AA is crucial for assessing exposure levels and health risks [1]. In 2017, the European Commission adopted Regulation (EU) 2017/2158, which established benchmark levels for specified food categories and required mitigation measures to reduce AA levels in food [10]. Moreover, common foods such as potatoes, bread, coffee, and snacks can contain varying concentrations of AA [11]. Consequently, reliable analytical methods are essential for meeting regulatory requirements and helping ensure consumer safety.

Currently, the detection of AA in foods is primarily based on chromatographic or mass spectrometric techniques, including gas chromatography (GC) [12], gas chromatography–mass spectrometry (GC-MS) [13,14,15,16,17], liquid chromatography (LC), and liquid chromatography–tandem mass spectrometry (LC-MS/MS) [18,19,20,21,22,23,24]. However, because AA is highly polar and has low volatility, GC-MS detection usually requires derivatization to increase its volatility. The most widely used approach is bromination [13,14,15]. Alternative derivatization strategies include xanthydrol derivatization [16] and silylation with N,O-bis(trimethylsilyl)trifluoroacetamide (BSTFA) [17]. These derivatization steps increase the complexity of sample preparation and may lead to analyte loss [21]. LC-MS/MS is more widely applied; however, owing to AA’s high polarity (water solubility: 215.5 g/100 mL) [2,12], it is weakly retained on conventional C_18_ columns and often co-elutes with other polar interferences from the sample matrix [6,21,24], causing severe matrix effects [25]. Moreover, achieving retention usually requires a high proportion of aqueous mobile phase, which is unfavorable for electrospray ionization (ESI) efficiency and thus reduces detection sensitivity [21,26]. This problem is particularly pronounced in complex matrices such as coffee, where AA analysis suffers from strong interference [27]. Several reports have noted an interfering peak eluting near AA [27,28], leading to overestimation of AA levels in coffee. Some researchers have investigated this interference in detail using liquid chromatography–high-resolution mass spectrometry (LC-HRMS) and attributed it to in-source fragmentation of N-acetyl-β-proline, which produces a fragment ion at *m*/*z* 72.044390 ([C_3_H_6_NO]^+^) that interferes with AA analysis in coffee; this fragment is isomeric to AA and cannot be distinguished by MS/MS or high-resolution mass spectrometry (HRMS) [27]. Therefore, achieving effective chromatographic separation is a viable strategy to circumvent this problem. To address the retention issues caused by AA’s high polarity, researchers have employed analytical methods using different types of chromatographic columns. First, hydrophilically modified C_18_ columns suitable for the analysis of polar compounds, such as Atlantis T3 [29] and Kinetex Polar C_18_ [30], have been used. Second, hydrophilic interaction liquid chromatography (HILIC) has been employed for separation [21,24]. Third, ion-exclusion chromatography has been used [31]. Fourth, separation has been performed using porous graphitic carbon (PGC) chromatography [18,32].

PGC is a unique packing material, composed of carbon atoms arranged in a hexagonal lattice, which can retain and separate highly polar compounds [33,34]. Its retention mechanisms mainly include adsorption and interactions between polar analytes and the carbon surface arising from charge polarization. The synergistic effects of multiple retention mechanisms confer higher selectivity of PGC packing for polar compounds and their isomers [33,35]. PGC columns are primarily used for the separation and analysis of polar compounds, including polysaccharides and glycans [36,37], peptides [38], and small polar molecules [33]. In the analysis of small molecules, PGC columns have been reported for the analysis of ultrashort-chain and short-chain per- and polyfluoroalkyl substances (PFAS) and have effectively separated them from matrix interferences [39], achieving excellent peak shapes and thereby improving detection sensitivity and accuracy. Similar to ultrashort-chain and short-chain PFAS, AA is highly polar and highly water-soluble, rendering PGC columns a feasible approach for the chromatographic separation of AA in foods.

Several reports have described the application of PGC for the analysis of AA. In 2007, Rosén et al. systematically compared the retention capacity of 14 SPE sorbents and 9 HPLC stationary phases for AA and found that the Hypercarb PGC column exhibited the strongest retention when water was used as the mobile phase, with a capacity factor of 4 [32]. Subsequently, several studies adopted PGC-based LC-MS/MS methods for the determination of AA in various food products [18,40]. European Standard EN 16618:2015 also recommends the use of PGC-based columns for AA analysis in food [41]. However, despite the application of PGC columns in AA analysis, the intrinsic matrix interference tolerance of PGC columns has rarely been rigorously evaluated, because the reported methods generally employ a two-step solid-phase extraction (SPE) procedure. The first step serves as a “chemical filtration” to remove matrix compounds using SPE cartridges that incorporate hydrophobic (C_18_), cation-exchange, and anion-exchange mechanisms; the second step uses an enrichment cartridge to retain AA before LC-MS/MS analysis [18,32,40,41]. This two-step procedure is time-consuming and, more importantly, such extensive cleanup removes most matrix components before the PGC column, thereby masking its intrinsic matrix tolerance and making it difficult to assess its ability to handle co-elution and matrix effects. Based on the strong retention of PGC for highly polar analytes, we hypothesize that a PGC-based LC-MS/MS method can effectively separate AA from co-eluting matrix interferences in complex food matrices, thereby reducing matrix effects and improving quantification accuracy compared with conventional reversed-phase columns. Therefore, the specific objective of this study was to rigorously evaluate the PGC column’s true interference resistance using a simplified, single-step mixed-mode cation-exchange (MCX) SPE cleanup method. Because MCX cartridges operate on a pass-through principle, most polar matrix components similar to AA remain in the sample solution, thereby providing a realistic and challenging environment in which to test the intrinsic separation capacity of the PGC column. We systematically compared the analytical performance of PGC and polar C_18_ columns for the determination of AA in meat products and coffee. Furthermore, the PGC-based LC-MS/MS analytical workflow was applied to analyze potato chips, biscuits, and local specialty foods, such as fried vinegar-marinated pork, Fuguo [oyster fritter], Cunzao [fried flour bar], and Malao [sesame-coated glutinous rice bar], to verify the applicability of the method to real-sample analysis and to provide a strategy for monitoring AA in foods.

## 2. Results

### 2.1. Comparison of Chromatographic Separation Using C_18_ and PGC

**Figure 1:**
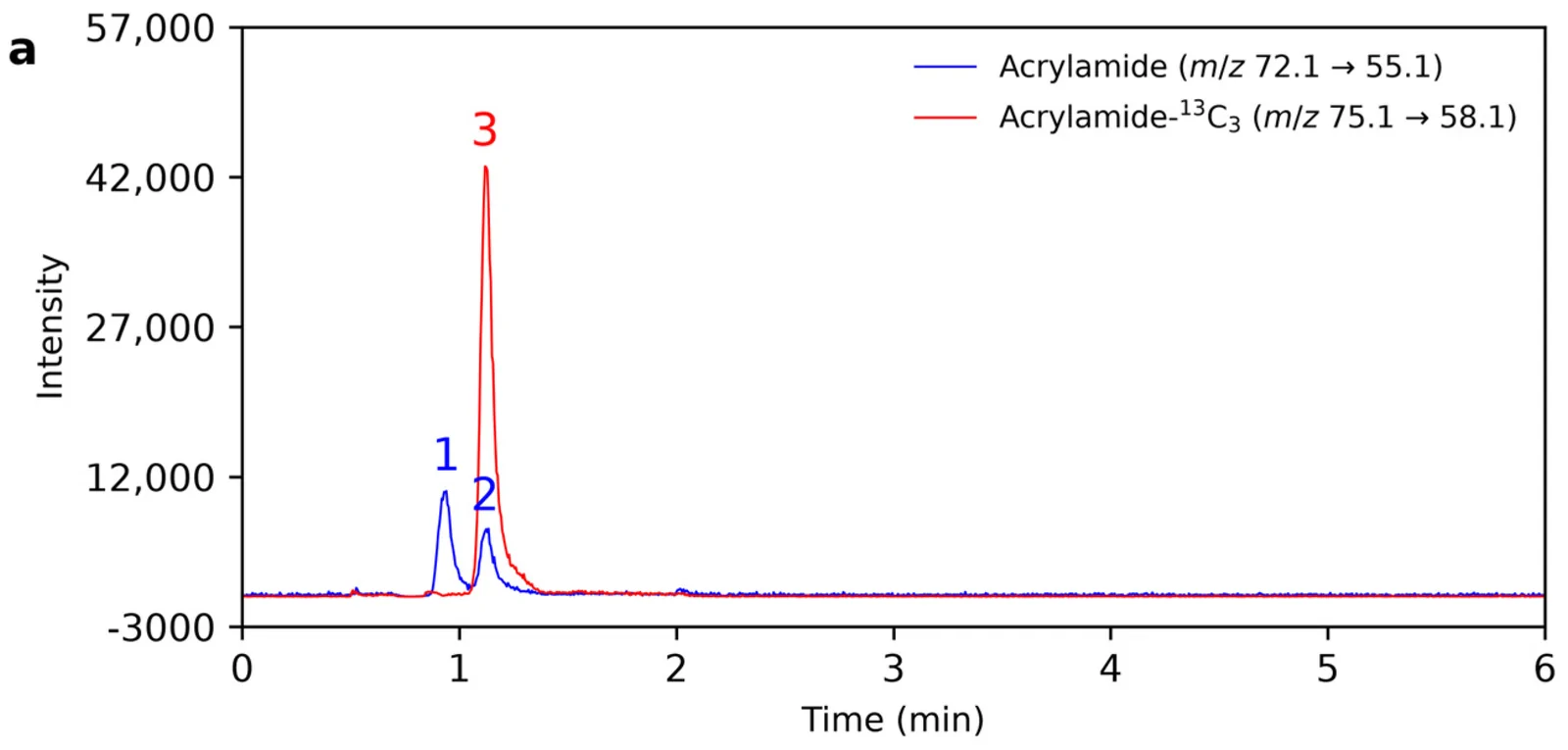

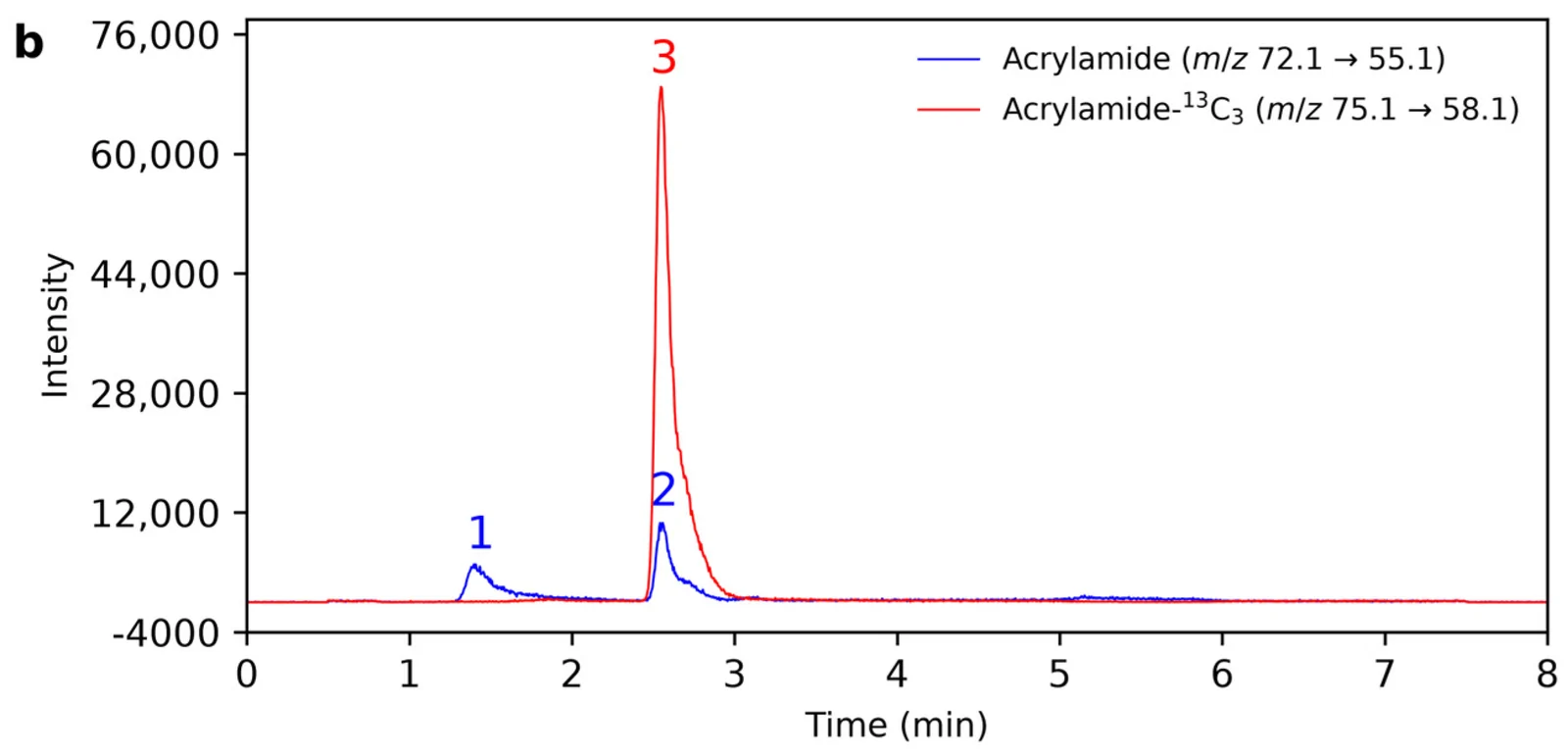
Extracted ion chromatograms of a meat-product matrix-matched standard solution (20 µg/L) obtained using columns as follows: (**a**) Polar C_18_ column, (**b**) PGC column. The blue and red traces correspond to acrylamide (*m*/*z* 72.1 → 55.1) and acrylamide-^13^C_3_ (*m/z* 75.1 → 58.1), respectively. Peaks: 1, interference; 2, acrylamide; 3, acrylamide-^13^C_3_.

AA is a small molecule with high water solubility and strong polarity. Traditional analyses use conventional C_8_ or C_18_ columns with highly aqueous mobile phases, but such columns exhibit weak retention for polar compounds [21,24]. To enhance the retention of polar compounds and enable the use of highly aqueous mobile phases, various polar-embedded stationary phases are commercially available. These stationary phases incorporate polar functional groups, such as amide, urea, carbamate, and ether groups [42], as well as other novel functional groups such as thioether and quaternary ammonium [43], into the octadecyl ligands near the silica surface, thereby increasing the hydrophilicity of the stationary phase and improving the retention of polar analytes. In this study, a Kinetex Polar C_18_ column was used. This column combines C_18_ ligands with a proprietary polar-group-modified surface. The exact chemical identities of these polar groups are proprietary to the manufacturer and have not been disclosed [44]. However, in practical applications, the column’s analytical performance for complex sample matrices remains unsatisfactory. Figure 1a shows the extracted ion chromatogram (EIC) of a matrix-matched standard solution (20 µg/L) in meat products, obtained using the Polar C_18_ column. The chromatogram indicates that AA exhibits weak retention on the column and poor separation from interfering peaks. Notably, the Polar C_18_ column uses smaller particles (2.6 μm); smaller particle sizes generally improve column efficiency, producing sharper peak shapes and higher sensitivity [45]. Nevertheless, the PGC column (3.0 μm) still provided significantly better retention, baseline resolution, and reduced matrix effects, further underscoring its unique separation mechanism for highly polar analytes.

It should be noted that the chromatographic separation parameters for the two columns (Section 3.5) were determined under conditions optimized for each column to achieve optimal separation of AA. Therefore, the comparison presented here reflects the analytical performance of the two chromatographic systems for AA under their respective optimal conditions, rather than the intrinsic retention capabilities of the stationary phases under identical mobile-phase conditions.

**Figure 2:**
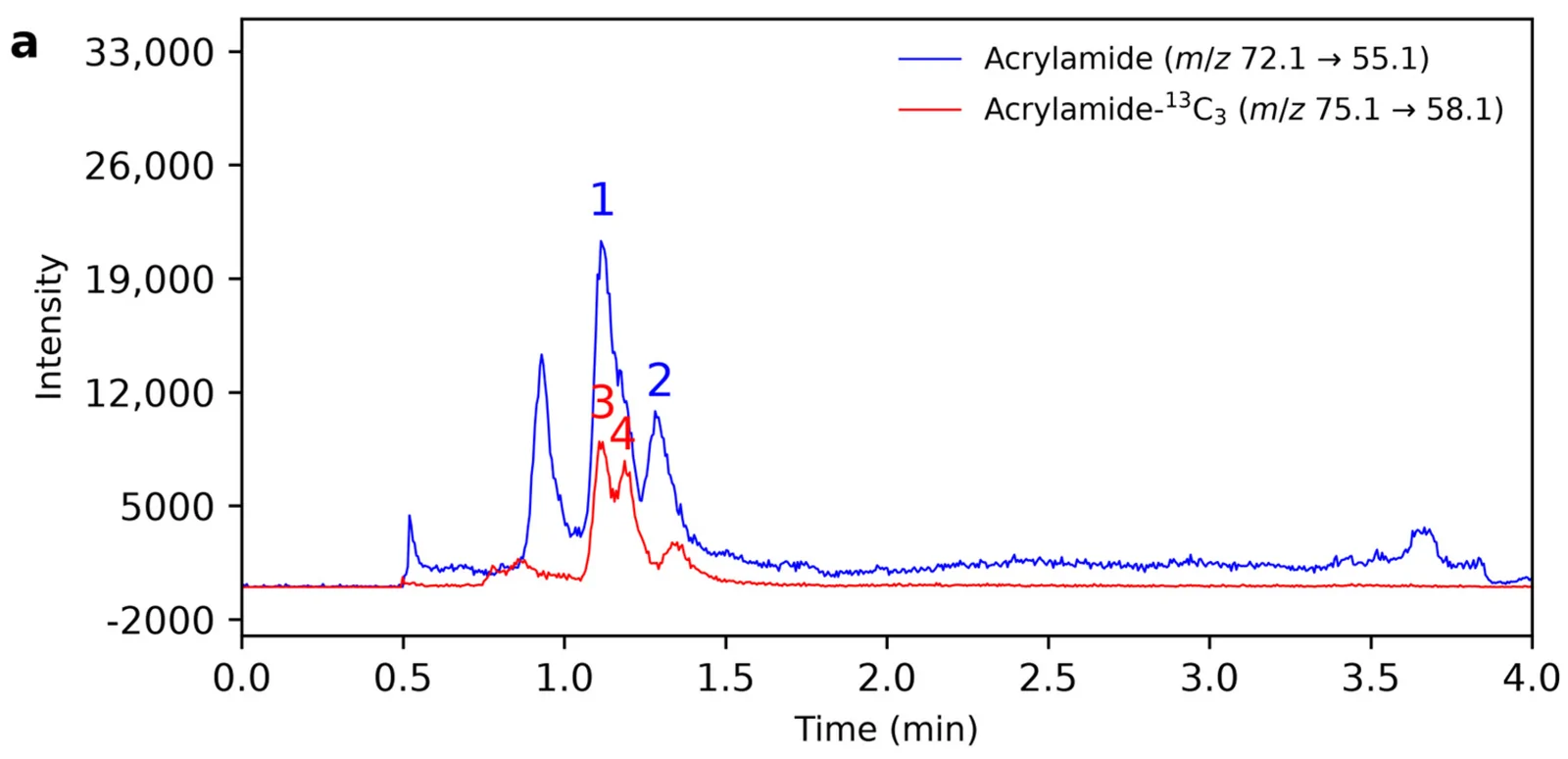

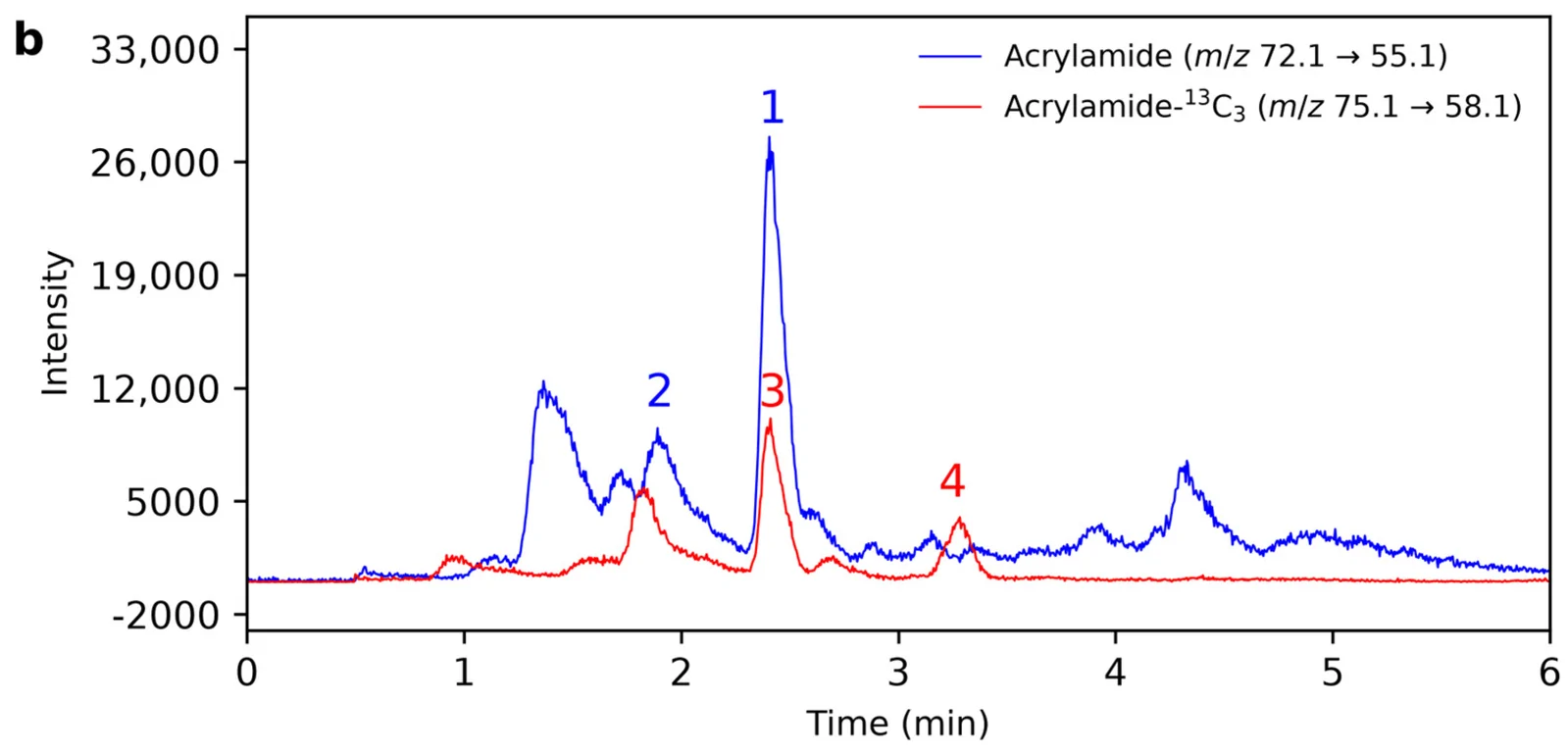
Extracted ion chromatograms for a coffee matrix after MCX solid-phase extraction, obtained using columns as follows: (**a**) a Polar C_18_ column (*S*/*N* of AA = 60.3:1), (**b**) a PGC column (*S*/*N* of AA = 90.1:1). The blue and red traces correspond to acrylamide (*m*/*z* 72.1 → 55.1) and acrylamide-^13^C_3_ (*m*/*z* 75.1 → 58.1), respectively. Peaks: 1, acrylamide; 2, interference; 3, acrylamide-^13^C_3_; 4, interference.

In addition to AA being easily affected by matrix components, its isotopically labeled internal standard in some samples can also suffer from matrix interference. Figure 2a shows the analytical chromatogram of a coffee sample; it can be seen that the AA peak and acrylamide-^13^C_3_ internal standard peak in the coffee sample purified with an MCX solid-phase extraction cartridge exhibit strong interference. This indicates that, despite the use of a modified C_18_ column, the high polarity of AA results in weak retention and ineffective separation from matrix components, which remains a challenge; simple solid-phase extraction purification methods are insufficient to remove the interference. Therefore, some studies have employed GCB/SCX/PSA mixed solid-phase extraction cartridges [46] or a two-step SPE procedure for the cleanup of AA in coffee [18].

PGC has a structure and retention characteristics that are markedly different from those of conventional bonded silica phases. It possesses a wide pH tolerance range and can effectively retain and separate highly polar compounds [34]. The retention mechanism of PGC involves multiple modes of interaction, including adsorption, interactions between polar analytes and the polarized carbon surface induced by charge, and a recognition process influenced by solute polarity and molecular planarity [33,34,35]. The planarity of a molecule is crucial to its retention on PGC, as planar molecules can align more closely with the graphite surface, thereby generating stronger interactions [33]. In addition, molecular simulation studies have explored the geometric configuration of π–π stacking interactions on PGC and identified these interactions as a factor contributing to retention on PGC. Conjugated analytes with extended planar conformations can interact with the graphite surface in a coplanar geometry [47]. AA possesses a planar conjugated structure (C=C–C(=O)–NH_2_), which enables maximum surface contact with the flat hexagonal carbon lattice of PGC through π–π stacking and charge-induced dipole interactions [48]—interactions that are unavailable on conventional alkyl-bonded C_18_ phases. These specific interaction mechanisms enable successful retention and separation of analytes that cannot be resolved by conventional reversed-phase HPLC, providing a new separation strategy for compounds that are challenging to analyze by reversed-phase chromatography. Figure 1b shows chromatograms of spiked meat product matrix solutions and coffee samples separated on a PGC column. As observed, analysis using the PGC column yields enhanced retention of the AA target peak and effective separation from interfering peaks, greatly reducing matrix interference caused by co-elution with other polar substances.

Compared with the C_18_ column, a higher proportion of organic solvents can be used (methanol increased from 2% to 30%), which significantly enhances electrospray ionization efficiency [26]. Moreover, the effective resolution on the PGC column minimizes co-eluting ion suppression, collectively leading to a substantial increase in response (the signal of the isotopically labeled internal standard increased from 40,000 to 69,000, an increase of 72.5%, at the same concentration), which can improve the sensitivity and accuracy of AA detection in complex matrices. Figure 2b presents the analytical chromatogram of a coffee sample after simple pretreatment with an MCX cartridge. When using a C_18_ column, the internal standard peak and interfering peaks are poorly resolved (Figure 2a), whereas good separation is obtained on the PGC column (Figure 2b). There is no need to use CARB/SCX/PSA solid-phase extraction cartridges for cleanup.

### 2.2. Optimization of Mass Spectrometry Parameters

The AA standard solution (1 mg/L) and the acrylamide-^13^C_3_ isotope-labeled internal standard solution were used to optimize the mass spectrometric parameters, including multiple reaction monitoring (MRM), declustering potential (DP), and collision energy (CE). First, the solutions were infused into the mass spectrometer using a syringe pump at a flow rate of 10 µL/min for precursor ion scanning. The ions with the highest intensities were selected as precursor ions (*m*/*z* 72.1 for AA and *m*/*z* 75.1 for acrylamide-^13^C_3_). By evaluating the effect of different DP voltages on the AA precursor ion, the optimal DP voltage was determined to be 50 V. Product ion scanning was then performed for the AA precursor ion at *m*/*z* 72.1, and the most intense product ion was selected. The main product ions were *m*/*z* 55.1 and 44.1, with *m*/*z* 55.1 showing the highest intensity; therefore, the transition *m*/*z* 72.1→55.1 was selected as the quantitative ion pair. This fragmentation involves the loss of an ammonia molecule (NH_3_, 17 Da) from the precursor ion, forming the acryloyl cation, [CH_2_=CH–C≡O]^+^ (*m*/*z* 55.1) [21]. The loss of an ethylene molecule (C_2_H_4_, 28 Da) from the precursor ion forms [H–N=C=O + H]^+^ (*m*/*z* 44.1), which was used as the confirmation ion. Similarly, acrylamide-^13^C_3_ (*m*/*z* 75.1) loses NH_3_ to form [^13^CH_2_=^13^CH–^13^C≡O]^+^ (*m*/*z* 58.1) [21]. This ion was used as the quantitative ion for the isotope-labeled internal standard. Under the selected MRM mode, the optimal CE was determined to be 16 V by testing a series of collision energies. Other parameters, such as nebulizer gas, heater gas, curtain gas, and collision gas, are automatically optimized by the instrument.

### 2.3. Optimization of the Mobile Phase

**Figure 3:**
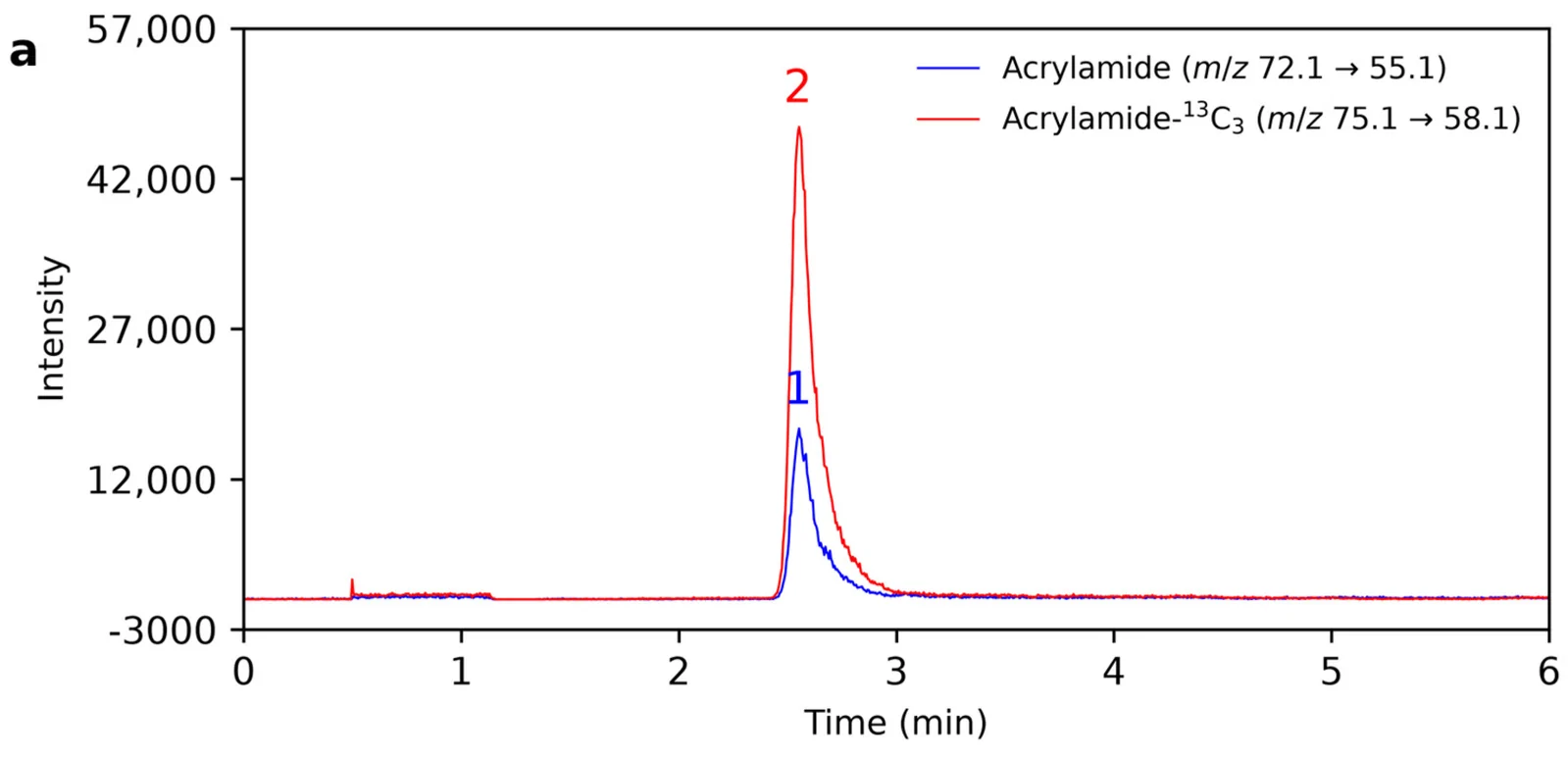

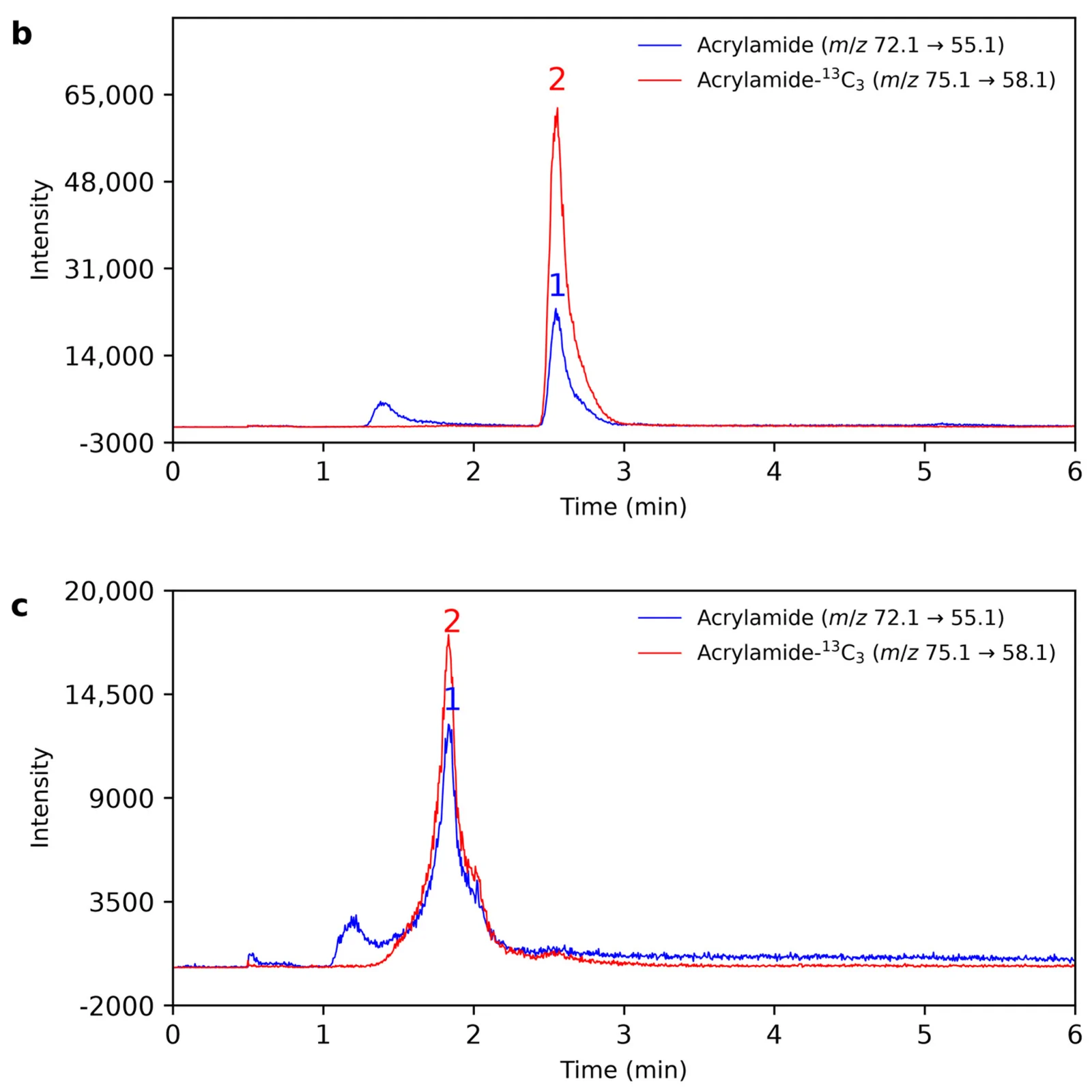
Extracted ion chromatograms of acrylamide and its internal standard, acrylamide -^13^C_3_, obtained using different mobile phase compositions: (**a**) water/methanol, (**b**) 0.1% formic acid in water/methanol, and (**c**) 0.1% formic acid in water/acetonitrile. The blue and red traces correspond to acrylamide (*m*/*z* 72.1 → 55.1) and acrylamide-^13^C_3_ (*m*/*z* 75.1 → 58.1), respectively. Peaks: 1, acrylamide; 2, acrylamide-^13^C_3_.

The effects of different mobile phases on AA analysis were compared using methanol–water (Figure 3a) and methanol–0.1% formic acid (Figure 3b). The results showed that the retention behavior of AA was not significantly affected. However, in ESI+ mode, 0.1% formic acid improved the ionization efficiency of AA, thereby enhancing the response intensity. Acetonitrile and methanol were also compared as organic modifiers. When acetonitrile was used, the AA peak shape was asymmetric (Figure 3c), whereas methanol provided better peak shape and response (Figure 3b). This may be attributed to methanol being a protic solvent with two lone pairs of electrons on oxygen, which can support hydrogen bonding and charge-induced dipole interactions with the PGC surface [49]. Therefore, water containing 0.1% formic acid and methanol were ultimately selected as the mobile phases.

### 2.4. Effect of Different Methanol Concentrations on Acrylamide Retention

**Figure 4:**
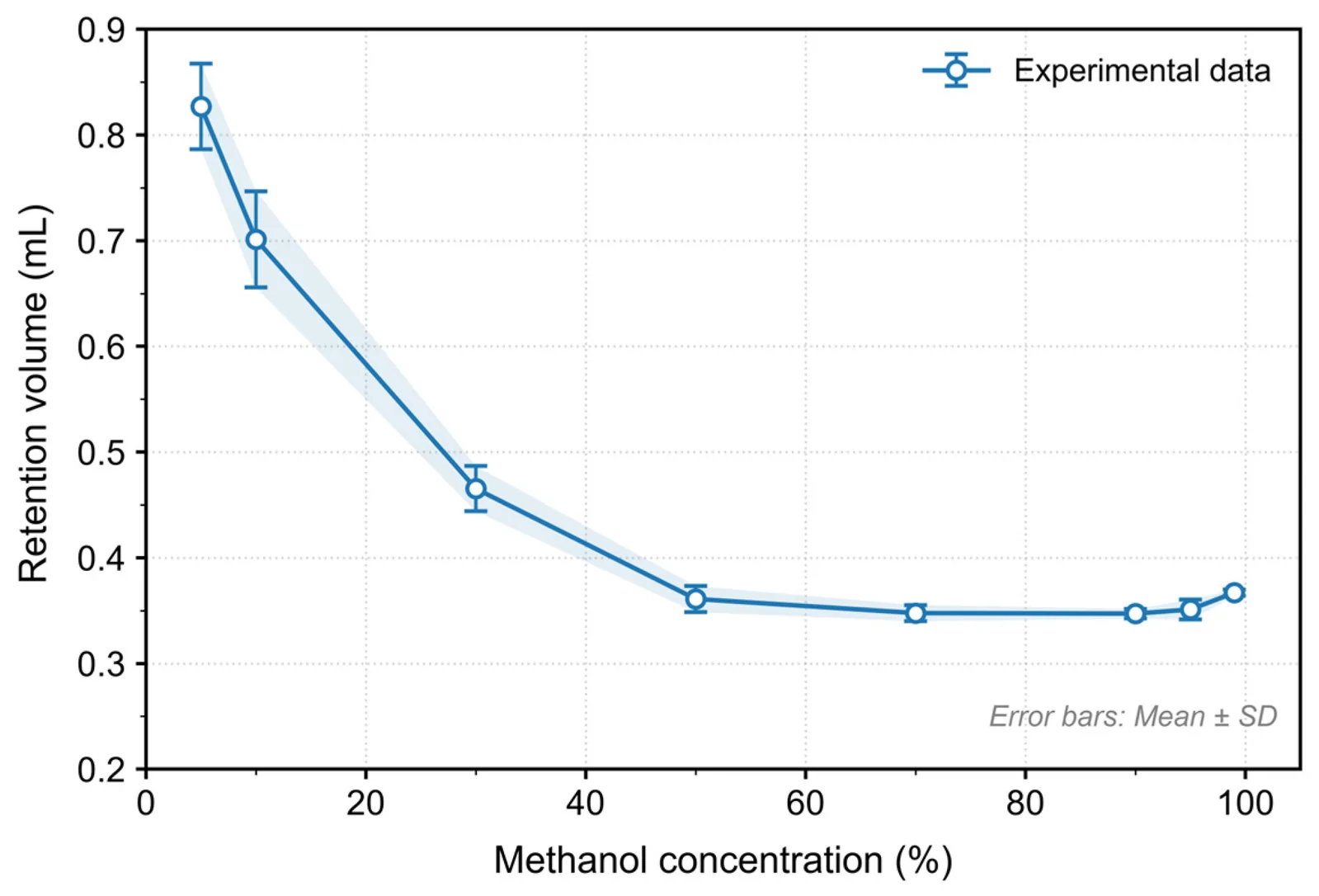
Retention volume of acrylamide on the PGC column at different methanol concentrations (5–99%). Data are presented as mean ± standard deviation (SD) of six independent measurements (*n* = 6),with the shaded area indicating the error band.

To investigate the retention behavior of AA on the PGC column under different methanol concentrations, isocratic elution was performed using methanol concentrations of 5%, 10%, 30%, 50%, 70%, 90%, 95%, and 99%, with Phase A maintained as 0.1% formic acid in water. Each concentration level was tested in six independent replicates (*n* = 6). As shown in Figure 4 (data are presented as mean ± SD), the retention volume of AA decreased with increasing methanol concentration from 5% to 90%, consistent with the fundamental principles of reversed-phase chromatography.

When the methanol proportion increased from 70% to 90%, the retention of AA changed little; however, unexpectedly, a further increase in the methanol (from 90% to 99%) led to an increase in AA retention, which was contrary to typical reversed-phase chromatographic behavior. Similar phenomena have also been reported when using PGC columns to analyze ultrashort-chain PFAS [39]. This may be attributed to the multiple retention mechanisms of PGC. A study by Lepont et al. demonstrated that, in a methanol–water mobile phase, cavity formation–dispersion interactions played a dominant role in the retention behavior of PGC, whereas the system constants for electron-pair interactions and dipole interactions varied nonlinearly, reaching their maximum and minimum values, respectively, at specific mobile-phase compositions [50]. This nonlinear dependence reflects the mobile-phase dependence of the multiple retention mechanisms of PGC. Tashinga et al. also examined how the mobile phase regulates the ionization states of both the analytes and the PGC surface and suggested that the mobile phase is the primary factor determining retention behavior [39,49].

Therefore, in the gradient elution design, low methanol concentration was initially used with a slow increase to maximize AA retention, followed by a ramp to 95% to efficiently elute strongly hydrophobic compounds.

### 2.5. Methodological Validation and Comparison

Method validation was performed using meat products and coffee samples. Meat products and coffee were selected because the main objective of this study was to evaluate the anti-interference capability of the PGC column in complex matrices. Coffee is a well-documented high-interference matrix, and meat products represent a high-protein/high-fat complex matrix. Because coffee samples free of AA are difficult to obtain for method validation, this study used unroasted green coffee, which was found to have an AA content below the detection limit, as the blank matrix. This surrogate approach has been previously reported in coffee analysis [51]. Matrix differences caused by roasting were partially compensated for by quantification using an isotopically labeled internal standard.

Blank meat product and coffee sample solutions were prepared according to Section 3.2, and calibration series were prepared in the corresponding blank matrix solutions. Linear regression was performed by plotting the peak area ratio of the quantifier ion to the isotopic internal standard (*Y*) against the concentration ratio (*X*). The method exhibited good linearity for AA over the concentration range of 10–500 µg/L. For meat products, the correlation coefficients for the C_18_ and PGC columns were 0.999 7 and 0.999 8, respectively; for coffee samples, the corresponding values were 0.999 4 and 0.999 7, respectively.

Recovery tests were conducted by spiking AA standard solutions into representative complex matrices (meat products and coffee) at final concentrations of 5.0, 50.0, and 200 µg/kg. At each spiked level, six independent replicates were prepared from separately weighed sample portions and subjected to independent extraction, purification, and injection. Mean recoveries and relative standard deviations (RSDs) were subsequently calculated. With the PGC column, mean recoveries for meat products ranged from 94.2% to 101.5% (RSDs of 1.5–5.4%), while those for coffee samples ranged from 100.5% to 103.4% (RSDs of 0.8–4.6%). Using the C_18_ column, recoveries for meat products ranged from 91.7% to 101.5%, with RSDs of 2.4–7.0%. However, analysis of spiked coffee samples using the C_18_ column yielded recovery rates ranging from 99.0% to 272.2%, with RSDs of 3.3–9.9%. Due to interference from co-eluting substances, the recoveries far exceeded the acceptable range. This finding is consistent with previous reports that traditional analytical methods may overestimate AA levels in coffee samples [27].

The limits of detection (LOD) and quantification (LOQ) were calculated from chromatograms of matrix-matched standards at low concentrations, using signal-to-noise ratios (*S*/*N*) of 3 and 10, respectively. For the PGC column, the LOD and LOQ for meat products were 0.81 and 2.70 µg/kg, respectively, and for coffee samples were 1.38 and 4.61 µg/kg. For the C_18_ column, the corresponding values for meat products were 1.95 and 6.49 µg/kg. Compared with the C_18_ column, the PGC column reduced the LOD for meat products from 1.95 to 0.81 µg/kg and the LOQ from 6.49 to 2.70 µg/kg, corresponding to a reduction of approximately 58.4% for both parameters. For coffee samples, the apparent LOD and LOQ obtained with the C_18_ column were 0.89 and 2.97 µg/kg, respectively. As discussed in Section 2.1, the PGC column provides higher sensitivity for AA analysis, which was confirmed in meat product analyses, where the PGC column yielded superior detection and quantification limits. However, when the C_18_ column was used to analyze coffee samples, co-eluting interferences with MRM transitions identical to those of AA artificially increased the *S/N*, resulting in anomalously low apparent LOD and LOQ values. This is consistent with the exceptionally high recoveries (up to 272%). Consequently, the fundamental limitation of the C_18_ column is its inability to reliably quantify AA in the coffee matrix. The LOD/LOQ estimates are misleading and do not reflect the method’s actual sensitivity for detecting low levels of AA. Therefore, to avoid presenting misleading data and ensure consistency with Table 1, these unreliable estimates have been removed (indicated by “—”) and are not considered valid parameters.

The method validation results were compared with the relevant regulatory and standard requirements. First, the validation results were compared with the AOAC Appendix F: Method Performance Requirements [52]. Appendix F, Table A5 provides target mean recovery ranges that broaden as analyte concentration decreases: for 100 µg/kg, the target range is 80–110%; for 10 µg/kg, the target range is 60–115% [52]. Table A4 provides target repeatability RSD values; at 10 µg/kg and 100 µg/kg, the RSDᵣ values are 21% and 15%, respectively [52]. Second, the validation results were compared with the AA-specific standard AOAC SMPR^®^ 2022.006, which specifies a recovery of 75–110% and repeatability of RSDᵣ = 0.66 × RSD_R (derived from the modified Horwitz equation) [53]. Regarding the LOQ, the standard specifies an LOQ ≤ 20 µg/kg for foods intended for infants and young children and bread, whereas for other foods, the LOQ must be ≤50 µg/kg [53]. Therefore, the method validation data based on the PGC column met the target performance criteria in Appendix F (Tables A4 and A5) and complied with the criteria specified in SMPR 2022.006 [53].

Matrix effects (ME) generally arise when matrix components in a sample co-elute with the analyte and suppress or enhance the ionization of the target analyte, thereby changing its response. Evaluating ME therefore provides, to some extent, an indication of the separation between matrix components and the analyte and thus reflects the stationary phase’s ability to separate the analyte from matrix components. ME are generally assessed by comparing the analyte response in a matrix solution with that in a pure solvent. However, when Polar C_18_ is used to analyze AA in coffee, matrix components in the sample co-elute with AA. Therefore, rather than using the AA response ratio, this study assessed ME using the response ratio of acrylamide-^13^C_3_.

As described in Section 3.3, a series of calibration solutions was prepared in pure water, and the ME was calculated as the ratio of the internal standard response obtained from a matrix-matched calibration curve to that derived from a solvent-based calibration curve. To exclude the effects of concentration differences and the matrix itself from the assessment of matrix effects for the two columns, a paired *t*-test was used. ME values were measured at six different concentration levels (10, 20, 50, 100, 200, and 500 µg/L), and the six ME values obtained using the C_18_ and PGC columns were paired. Statistical comparisons were performed using a paired *t*-test. For meat products, the ME values for the C_18_ and PGC columns were 46.8% and 76.5%, respectively; for coffee samples, they were 51.3% and 60.6%, respectively. The ME values of AAs obtained using the PGC and C_18_ columns were compared using a paired *t*-test. The paired differences were first tested for normality using the Shapiro–Wilk test (for coffee samples: *W* = 0.931, *p* = 0.588), indicating no significant departure from normality. For meat products, the Shapiro–Wilk test (*W* = 0.79, *p* = 0.051) also indicated no significant departure from normality, although the result was borderline. The paired *t*-test showed a statistically significant difference in ME between the two columns (for coffee: *t* (5) = 4.62, *p* = 0.0057; for meat products: *t* (5) = 20.05, *p* < 0.0001). These results indicate that the PGC column exhibits higher ME values, corresponding to a smaller extent of signal suppression, compared with the C_18_ column in the analysis of coffee and meat products. However, it should be noted that this statistical significance is limited strictly to the matrices and experimental conditions evaluated in this study. This difference between the two columns can be attributed to the weaker retention of AA on the C_18_ column, a phenomenon that readily leads to co-elution with polar matrix components. In contrast, the PGC column can more effectively separate AA from interfering components, thereby reducing signal suppression caused by the matrix.

A comprehensive methodological comparison of the C_18_ and PGC columns for AA analysis in meat products and coffee samples is presented in Table 1.

As mentioned in the Introduction, current approaches for addressing the poor retention of AA in C_18_ chromatography mainly include modified C_18_ columns [29,30], HILIC [21,24], PGC [18], and ion-exclusion chromatography [31]. The PGC method employed in this study is compared with several other representative methods reported in the literature, as shown in Table 2.

### 2.6. Determination of Real Samples

The established method was applied to analyze actual samples of different matrices; the real samples included both local specialty foods from southern Fujian and commercially available products. It should be noted that full method validation was conducted in meat products and coffee, whereas its application to other sample categories was intended to demonstrate the method’s practical utility. Specifically, four types of local specialty foods were analyzed: Fried vinegar-marinated pork (*n* = 20, 3.05–27.7 µg/kg), Fuguo [oyster fritter] (*n* = 10, 11.8–37.9 µg/kg), Cunzao [fried flour bar] (*n* = 10, 17.7–174.5 µg/kg), and Malao [sesame-coated glutinous rice bar] (*n* = 10, 9.05–61.8 µg/kg). In addition, roasted coffee beans (*n* = 3, 227.4–648.4 µg/kg), instant coffee (*n* = 3, 24.5–78.4 µg/kg), biscuits (*n* = 6, 85.3–542.5 µg/kg), and potato chips (*n* = 6, 51.1–552.2 µg/kg) were also analyzed.

To provide an indicative assessment of the AA levels in the collected samples, the measured contents were compared with the benchmark levels established by Commission Regulation (EU) 2017/2158 (Table 3). This comparison does not constitute a formal regulatory classification. These benchmark levels serve as performance indicators to verify the effectiveness of mitigation measures, rather than legally enforceable maximum limits. For roasted coffee beans, the mean content (422.9 µg/kg) was slightly higher than the benchmark value of 400 µg/kg, whereas the mean content in instant coffee (58.5 µg/kg) was considerably lower than the benchmark value of 850 µg/kg. The mean content in biscuits was 228.1 µg/kg, below the benchmark value of 350 µg/kg. The mean content in potato chips was 334.3 µg/kg, below the benchmark value of 750 µg/kg. Because Fuguo, Cunzao, and Malao are local specialty fried snacks without a directly corresponding category in the regulation, the 300 µg/kg benchmark for “products similar to other products in this category” was used merely as an indicative reference. Their mean contents of 21.2, 71.4, and 34.8 µg/kg, respectively, are well below this reference level. Fried vinegar-marinated pork is a starch-coated fried meat product, for which the Regulation does not specify a corresponding benchmark level.

Regarding the LOQ, the regulation stipulates that where the benchmark level is <125 µg/kg, the LOQ must be ≤two-fifths of the benchmark level; for a benchmark level ≥125 µg/kg, the LOQ must be ≤50 µg/kg [10]. The LOQs obtained in this study (2.70–4.61 µg/kg) are well below the 50 µg/kg requirement and satisfy the strictest two-fifths criterion, confirming that the method is sufficiently sensitive for regulatory monitoring. The measured concentrations in roasted coffee samples were consistent with previously reported levels in the literature [27], further confirming the applicability of the proposed method.

The quantitative results for all real samples are summarized in Table 3.

### 2.7. Limitations, Future Research Directions, and Applications

Although the PGC-based method presented in this study offers advantages for improving AA retention and separation, several limitations should nevertheless be acknowledged. First, the method validation focused on meat products and coffee, whereas other AA-containing foods, such as infant foods, cocoa products, and cereals, were not included in the study. Second, green coffee was used as a surrogate blank matrix for roasted coffee. Because roasting generates Maillard products and alters the matrix composition, green coffee cannot fully represent roasted coffee. Third, although the method was applied to determine eight sample categories (*n* = 68), the sample sizes for certain matrices, such as roasted coffee beans and instant coffee (*n* = 3 each), were relatively small, which may limit the statistical robustness of the conclusions for these matrices. Fourth, although the PGC column exhibited a higher ME value (60.6–76.5%) than the C_18_ column (46.8–51.3%), indicating a smaller extent of signal suppression, the residual matrix effect was not completely eliminated; While the isotope-labeled internal standard can effectively correct for this signal suppression within the validated matrices (meat products and coffee), the effectiveness of this internal standard correction requires further validation in other uninvestigated food matrices. Fifth, the statistical significance of the matrix effect differences observed in this study (*p* < 0.05) is specific to the tested matrices. Future research should further evaluate the statistical applicability of these findings across a broader range of complex food matrices.

Future research could address the mentioned limitations in the following directions. First, the sample size for each matrix should be increased, and more AA-containing food matrices, such as infant foods, cocoa products, cereals, and tea, should be included to further validate the broad applicability of the method. Second, inter-laboratory validation and inter-day precision studies should be conducted to evaluate the robustness of the method across different laboratories and over time. Third, the PGC-based separation strategy could be coupled with high-resolution mass spectrometry (HRMS) to further confirm the identities of co-eluting interferents and investigate the formation and mitigation of AA under different food-processing conditions. Fourth, the method could be extended to the simultaneous determination of other polar food-processing contaminants, such as 5-hydroxymethylfurfural, which likewise exhibit weak retention on conventional reversed-phase columns.

In terms of practical applications, the developed method is suitable for the routine monitoring of AA in complex food matrices, particularly coffee and meat products, because conventional C_18_-based LC–MS/MS methods are susceptible to co-eluting interferences, which can lead to overestimation. This PGC-based method provides a practical approach.

## 3. Materials and Methods

### 3.1. Chemicals and Reagents

AA standard solution (1000 µg/mL in methanol, Anpu Technology, Shanghai, China); acrylamide-^13^C_3_ internal standard (100 µg/mL in methanol, Anpu Technology, Shanghai, China); extraction salt packet (1.5 g sodium acetate and 6 g anhydrous magnesium sulfate, Anpu Technology, Shanghai, China); methanol (LC-MS grade, Merck, Darmstadt, Germany); formic acid 99% (HPLC grade, ROE SCIENTIFIC INC., Newark, DE, USA); acetonitrile and n-hexane (HPLC grade, TEDIA, Fairfield, OH, USA); ultrapure water.

### 3.2. Test Sample Information

The method-validation samples and test samples used in this study were purchased from JD.com, local supermarkets, and local on-site frying shops. Specifically, raw coffee beans (*n* = 2, used for method validation) and roasted coffee beans (*n* = 3) were purchased from JD.com; potato chips (*n* = 6), biscuits (*n* = 6), and instant coffee (*n* = 3) were purchased from local supermarkets. The local specialty foods included fried vinegar-marinated pork (a starch-coated fried meat product from southern Fujian, made by coating vinegar-marinated pork with sweet-potato starch and deep-frying it; *n* = 20), Fuguo [oyster fritter] (a traditional fried snack from southern Fujian, made by wrapping fillings such as oysters, lean pork, and shredded pumpkin in sweet-potato starch and deep-frying them; *n* = 10), Malao [sesame-coated glutinous rice bar] (a traditional tea snack from southern Fujian, made by deep-frying a dough prepared from glutinous rice and taro and then coating it with sesame or peanut powder; *n* = 10), and Cunzao [fried flour bar] (a traditional festive sweet from southern Fujian, made by deep-frying strips prepared from glutinous rice flour and then coating them with sugar; *n* = 10). All four local specialty foods were purchased from local on-site frying shops, where these foods were freshly prepared. Specific brand information was omitted because it was not relevant to this study.

### 3.3. Sample Preparation

The extraction and purification procedures were conducted in accordance with the GB 5009.204-2025 standard [22]. A homogenized sample of 2.00 g was accurately weighed, and 25 µL of the internal standard working solution (4.0 µg/mL) was added. The mixture was allowed to stand for 30 min to ensure complete penetration of the internal standard. Subsequently, 9 mL of water was added, and the sample was dispersed by vortexing. After an additional 30 min of shaking, the mixture was centrifuged at 4000 rpm for 10 min. The supernatant was then mixed with 5 mL of n-hexane, extracted by shaking for 5 min, and further centrifuged at 10,000 rpm for 5 min to separate and discard the organic phase. Then, 10 mL of acetonitrile was added to the aqueous extract and shaken for 10 min. Subsequently, extraction salt was added as a salting-out agent, and the mixture was shaken for another 5 min. After centrifugation at 10,000 rpm at 4 °C for 10 min, 3 mL of the acetonitrile layer was collected and passed through an MCX solid-phase extraction column. The eluate was collected and evaporated to dryness under a nitrogen stream. The residue was redissolved in 0.3 mL of water, filtered through a 0.22 μm aqueous filter membrane, and then subjected to LC-MS/MS analysis.

### 3.4. Preparation for Standard Solutions

Appropriate volumes of the AA standard solution and the acrylamide-^13^C_3_ internal standard solution were taken and sequentially diluted with water to obtain a 1 µg/mL analytical standard solution and a 4.0 µg/mL internal standard working solution. A series of calibration standards at concentrations of 10, 20, 50, 100, 200, and 500 µg/L were then prepared in water, with the concentration of the acrylamide-^13^C_3_ internal standard fixed at 100 µg/L.

### 3.5. Analysis Conditions

AA analysis was performed using an AB Sciex 4000+ triple quadrupole mass spectrometer (Sciex, Framingham, MA, USA) coupled to a 1290 UHPLC system (Agilent Technologies, Santa Clara, CA, USA) equipped with a binary high-pressure pump, an autosampler, and a column oven. Separations were conducted on a Hypercarb HT column (100 × 2.1 mm, 3 μm; Thermo Fisher Scientific, Waltham, MA, USA) and a Kinetex^®^ Polar C_18_ column (100 × 2.1 mm, 2.6 μm; Phenomenex, Torrance, CA, USA) under the following respective conditions.

For the Hypercarb HT column, mobile phase A was 0.1% formic acid in water and mobile phase B was methanol. The gradient elution program was as follows: 0–3.0 min, B ramped from 5% to 50%; 3.0–3.5 min, B ramped to 95% and held for 0.5 min (until 4.0 min); and 4.0–4.5 min, the mobile phase returned to the initial composition and was equilibrated for 3.5 min. The column temperature was 40 °C, the flow rate was 0.25 mL/min, and the injection volume was 5.0 µL.

For the Kinetex Polar C_18_ column, mobile phase A was 0.1% formic acid in water and mobile phase B was methanol. The gradient was as follows: 0–2.0 min, B held at 2%; 2.0–2.5 min, B ramped to 40%; 2.5–3.0 min, B ramped to 95% and held for 2 min (until 5.0 min); and 5.0–5.5 min, the mobile phase returned to the initial conditions and was equilibrated for 4.5 min. The column temperature was 40 °C, the flow rate was 0.3 mL/min, and the injection volume was 5.0 µL.

Mass spectrometry was performed in positive electrospray ionization (ESI+) mode. The source parameters were set as follows: nebulizer gas, 35 psi; heater gas, 35 psi; curtain gas, 50 psi; collision gas, 4 psi; and ion source temperature, 500 °C. The declustering potential (DP) and collision energy (CE) were set at 50 V and 16 V, respectively. Multiple reaction monitoring (MRM) was employed. The monitored ion transitions were *m*/*z* 72.1 →55.1 as the quantifier ion and *m*/*z* 72.1 →44.1as the qualifier ion for AA, and *m*/*z* 75.1 →58.1 for the internal standard (acrylamide-^13^C_3_). Data acquisition and quantitative analysis were performed using Analyst software (1.6.2, Sciex) and MultiQuant software (version 3.0, Sciex), respectively.

### 3.6. Preparation of Figures and Statistical Analysis

Extracted ion chromatograms (EICs, Figure 1, Figure 2 and Figure 3) and the point-and-line plot (Figure 4) were plotted using Python (version 3.14) with the Matplotlib library (version 3.11.2), at a resolution of 600 dpi. The raw mass spectrometry data were exported as text files using the “Data to txt” function in PeakView software (version 2.2), with the original retention times and response intensities preserved during the export process.

Statistical tests for matrix effects were performed using SAS software (version 9.4; SAS Institute, Cary, NC, USA). First, the Shapiro–Wilk test was used to assess normality. Subsequently, a paired *t*-test was employed to compare the ME values obtained using the two chromatographic columns. A *p*-value < 0.05 was considered statistically significant.

## 4. Conclusions

An LC-MS/MS method for the analysis of AA in food was established based on a PGC column. By employing a single-step pass-through MCX clean-up, the inherent anti-interference capacity of the PGC column in complex matrices was evaluated. The PGC column provides strong retention for polar compounds, thereby improving the chromatographic separation of AA from co-eluting interferences and reducing the overestimation issues commonly encountered on reversed-phase columns. Compared to traditional LC-MS/MS using C_18_ columns, this approach provides improved sensitivity in matrices with well-controlled interferences (e.g., meat products) and effectively prevents severe overestimation in complex matrices like coffee. Although a residual matrix effect (60.6–76.5%) was observed in complex matrices, its impact was effectively corrected by the isotope-labeled internal standard specifically within the validated matrices (meat products and coffee), supporting reliable quantification for these specific matrices. While statistically significant differences in matrix effects were observed between the columns, this significance is strictly limited to the specific matrices and experimental conditions evaluated in the present study. Therefore, this PGC-based method offers a practical approach for routine monitoring of AA strictly in these validated complex food matrices.

## Figures and Tables

**Table 1:** Methodological comparison of C_18_ and PGC columns for acrylamide analysis in meat products and coffee samples.

Matrix	Column	Linear Equations	r	LOD	LOQ	Recovery	RSD	Matrix Effects *
				(µg/kg)	(µg/kg)	%	%	%
Meats	C_18_	*Y* = 0.00703*X* + 0.002787	0.9997	1.95	6.49	91.7–101.5	2.4–7.0	46.8
	PGC	*Y* = 0.00722*X* − 0.00182	0.9998	0.81	2.70	94.2–101.5	1.5–5.4	76.5
Coffees	C_18_	*Y =* 0.00783*X* − 0.00376	0.9994	—^#^	—^#^	—^#^	3.3–9.9	51.3
	PGC	*Y =* 0.00764*X* + 0.02183	0.9997	1.38	4.61	100.5–103.4	0.8–4.6	60.6

# For coffee samples analyzed with the C_18_ column, co-eluting interferences produced artifactually elevated *S*/*N* and unreliable recovery (up to 272%); therefore, these apparent estimates were not considered valid method parameters. * A higher matrix effect (ME) value indicates a higher response ratio and less signal suppression.

**Table 2:** Comparison of published LC–MS/MS methods employing different separation strategies with the proposed method.

Stationary Phase	Column	Mobile Phase	Sample Preparation	LOD (µg/kg)	LOQ (µg/kg)	Recovery (%)	RSD (%)	ME (%)	Reference
Modified C_18_	Kinetex Polar C_18_	methanol + 0.1% acetic acid	Oasis HLB cartridge Bond Elut AccuCAT SPE column	1.86	5.62	N.R.	N.R.	N.R.	[30]
HILIC	Thermo Scientific Accucore HILIC	0.1% FA in water/0.1% FA in ACN	QuEChERS Mix I, QuEChERS Mix VI	2.71	9.02	100.5–102.8	2.7–3.6	−50 (ESTD); −12 (ISTD)	[21]
Ion exclusion	IonPac ICE-AS1 ion-exclusion column	FA in water/0.1% FA in ACN	Isolute activated carbon cartridges	23 pg (instrumental) ^a^	75 pg (instrumental) ^a^	97.6–143	6.3–13.0	Not reported	[31]
HILIC	TSKgel Amide-80 column	1 mM ammonium formate + 0.1% FA/ACN	Strata-XL-C SPE cartridges	8	20	101–105	2.9–7.6	66 for coffee	[24]
PGC	Hypercarb HT column	0.1% FA in water/methanol	MCX solid phase extraction column	0.81–1.38	2.70–4.61	94.2–103.4	0.8–5.4	60.6–76.5	(This paper)

LOD, limit of detection; LOQ, limit of quantification; FA, Formic acid; CAN, Acetonitrile; PGC, porous graphitic carbon; SPE, solid-phase extraction; MCX, mixed-mode cation exchange; N.R., Not Reported. ME, matrix effect (A higher ME value indicates a higher response ratio and less signal suppression). Note: This comparison is intended to be indicative only, as the reported methods were performed using different food matrices, sample preparation procedures, and analytical conditions. ^a^: The instrument detection limit is reported in picograms (pg).

**Table 3:** Acrylamide concentrations in real samples (n, range, mean, and standard deviation).

Sample	*n*	Range (µg/kg)	Mean (µg/kg)	SD (µg/kg)	EU Benchmark (µg/kg)
Fried vinegar-marinated pork *	20	3.05–27.7	9.6	6.4	— ^a^
Fuguo [oyster fritter] *	10	11.8–37.9	21.2	7.8	300 ^b^
Cunzao [fried flour bar] *	10	17.7–174.5	71.4	56.3	300 ^b^
Malao [sesame-coated glutinous rice bar] *	10	9.05–61.8	34.8	17.8	300 ^b^
Roasted coffee beans	3	227.4–648.4	422.9	212.1	400
Instant coffee	3	24.5–78.4	58.5	29.6	850
Biscuits	6	85.3–542.5	228.1	175.9	350
Potato chips	6	51.1–552.2	334.3	196.8	750

Note: The comparison with EU benchmark levels is provided as an indicative assessment of the tested samples and does not constitute a formal regulatory classification. Full method validation was performed only on meat products and coffee. EU benchmark levels are specified in Commission Regulation (EU) 2017/2158. These values serve as performance indicators to verify the effectiveness of mitigation measures, rather than legally enforceable maximum limits. *: Specialty foods from the southern Fujian region; refer to Section 3.2. ^a^: No corresponding category is specified in Regulation (EU) 2017/2158 for starch-coated fried meat products. ^b^: No directly corresponding category is specified in the regulation. The 300 µg/kg benchmark for “Products similar to the other products in this category” is provided solely as an indicative reference based on their starch-based matrix and deep-frying processing. This does not constitute a formal regulatory classification.

## Data Availability

The original contributions presented in this study are included in the article. Further inquiries can be directed to the corresponding author(s).

## Abbreviations

The following abbreviations are used in this manuscript:

AA	Acrylamide
PGC	Porous graphitic carbon
GC	Gas chromatographic
GC-MS	Gas chromatographic-mass spectrometry
LC-MS/MS	Liquid chromatography–tandem mass spectrometry
HRMS	High-resolution mass spectrometry
SPE	Solid-phase extraction
MCX	Mixed-mode cation exchange
QuEChERS	Quick, Easy, Cheap, Effective, Rugged, Safe
MRM	Multiple reaction monitoring
LOD	Limits of detection
LOQ	Limits of quantification
ME	Matrix effect
RSDᵣ	Relative standard deviation of repeatability
RSD_R_	Relative standard deviation of reproducibility
